# Metabolic reprogramming of stromal fibroblasts by melanoma exosome microRNA favours a pre-metastatic microenvironment

**DOI:** 10.1038/s41598-018-31323-7

**Published:** 2018-08-27

**Authors:** Shin La Shu, Yunchen Yang, Cheryl L. Allen, Orla Maguire, Hans Minderman, Arindam Sen, Michael J. Ciesielski, Katherine A. Collins, Peter J. Bush, Prashant Singh, Xue Wang, Martin Morgan, Jun Qu, Richard B. Bankert, Theresa L. Whiteside, Yun Wu, Marc S. Ernstoff

**Affiliations:** 1 Department of Medicine, Roswell Park Comprehensive Cancer Center, Buffalo, NY USA; 2 Department of Biomedical Engineering, Jacobs School of Medicine & Biomedical Sciences, University at Buffalo, The State University of New York, Buffalo, NY USA; 3 Flow and Image Cytometry Shared Resource, Roswell Park Comprehensive Cancer Center, Buffalo, NY USA; 4 Department of Cell Stress Biology, Roswell Park Comprehensive Cancer Center, Buffalo, NY USA; 5 Department of Neurosurgery, Roswell Park Comprehensive Cancer Center, Buffalo, NY USA; 6 Immune Analysis Facility, Center for Immunotherapy, Roswell Park Comprehensive Cancer Center, Buffalo, NY USA; 7 South Campus Instrumentation Center, University at Buffalo, The State University of New York, Buffalo, NY USA; 8 Genomics Shared Resource, Roswell Park Comprehensive Cancer Center, Buffalo, NY USA; 9 New York Center of Excellence in Bioinformatics and Life Sciences, Buffalo, NY USA; 10 Department of Biostatistics and Bioinformatics, Roswell Park Comprehensive Cancer Center, Buffalo, NY USA; 11 Department of Microbiology and Immunology, Jacobs School of Medicine & Biomedical Sciences, University at Buffalo, The State University of New York, Buffalo, NY USA; 12 Department of Pathology, Immunology and Otolaryngology, University of Pittsburgh School of Medicine and UPMC Hillman Cancer Center, Pittsburgh, PA USA

## Abstract

Local acidification of stroma is proposed to favour pre-metastatic niche formation but the mechanism of initiation is unclear. We investigated whether Human Melanoma-derived exosomes (HMEX) could reprogram human adult dermal fibroblasts (HADF) and cause extracellular acidification. HMEX were isolated from supernatants of six melanoma cell lines (3 BRAF V600E mutant cell lines and 3 BRAF wild-type cell lines) using ultracentrifugation or Size Exclusion Chromatography (SEC). Rapid uptake of exosomes by HADF was demonstrated following 18 hours co-incubation. Exposure of HDAF to HMEX leads to an increase in aerobic glycolysis and decrease in oxidative phosphorylation (OXPHOS) in HADF, consequently increasing extracellular acidification. Using a novel immuno-biochip, exosomal miR-155 and miR-210 were detected in HMEX. These miRNAs were present in HMEX from all six melanoma cell lines and were instrumental in promoting glycolysis and inhibiting OXPHOS in tumour cells. Inhibition of miR-155 and miR-210 activity by transfection of miRNA inhibitors into HMEX reversed the exosome-induced metabolic reprogramming of HADF. The data indicate that melanoma-derived exosomes modulate stromal cell metabolism and may contribute to the creation of a pre-metastatic niche that promotes the development of metastasis.

## Introduction

A high rate of cancer mortality (>90%) is associated with metastasis of primary tumour to distal organs. Among metastatic cancers, melanoma is the most lethal, with stage IV melanoma patients having a 5-year survival rate of less than 15%^1^. There is now a better appreciation that metastasis is intricately linked to the tumour microenvironment (TME) that not only allows tumour cell extravasation and circulation but helps to create a pre-metastatic niche in distal regions to aid in the implantation and survival of tumour cells^2–5^. The TME is continuously conditioned by the tumour to sustain immunosuppressive, inflammatory and metabolic activities of stromal cells in support of its invasive characteristics^6^. Nevertheless, some preclinical models continue to overlook the critical influence of the TME on metastasis^7,8^, discounting or minimising the role of the TME in clinical interventions, such as chemotherapy-induced metastasis^9^ and TME-assisted metastasis^10–12^. Increased extracellular acidification of the TME from glycolysis-driven metabolism^13^, known as the “Warburg effect”^14^, places an enormous burden on the immune response leading to anergy or incapacitation of T lymphocytes^15^. As observed in an *in vivo* melanoma metastasis study, acidic extracellular pH promotes metastasis and systemic correction of pH is sufficient to inhibit spontaneous metastases^16,17^. Likewise, initial acidification of the local milieu is proposed as a prognostic marker for a metastasis-permissive, pre-metastatic microenvironment^18^.

Despite many advances in understanding the cellular and molecular interactions that occur within the TME, the underlying mechanism contributing to the generation of the pre-metastatic niche at distal sites remains elusive. Emerging evidence suggests that cancer-derived extracellular vesicles (EVs) play a major role in not only conditioning the TME but also preparing the “soil” in the pre-metastatic niche for metastasis^5,19^. There are several types of EVs in the TME: microvesicles (MVs), apoptotic bodies and exosomes. In contrast to larger MVs and apoptotic bodies, exosomes are small (30–150 nm) membrane-bound vesicles that originate from multivesicular bodies (MVBs) through endosomal packaging. Exosomes released into extracellular space serve an essential role in cell-to-cell communication *via* the biologically-active payload that they carry, including proteins, lipids and metabolites as well as RNA and DNA species^20–23^. An example of this communication is seen in melanoma exosomes that can travel to distal regions to recruit bone-marrow derived cells to promote a pre-metastatic niche and predispose the site for metastasis^5^. In melanoma, BRAF mutation is a central driver in cancer and has led to the evaluation of BRAF inhibitors being evaluated in clinical trials. Exosomes derived from a BRAF (V600) mutation have been reported to harbor a different payload compared to exosomes from wild-type BRAF melanoma cells^24^.

Normal stromal cells such as fibroblasts play a critical role in inhibiting early-stage melanoma development^25^. Over time, such tumor suppressing activity of fibroblasts is lost through the influence of TME and these TME-conditioned fibroblasts instead induce increased tumorigenesis^26^ and metastasis^27^. Therefore identifying elements within the TME that conditions the stroma and revoking their influence is an attractive therapeutic intervention strategy for preventing pre-metastatic niche formation^12^. A human adult dermal fibroblast (HADF) cell line is an *in vitro* model for studying potential effects mediated by human melanoma exosomes (HMEX). Using normal HADF, we modeled the effects of the fibroblast-rich stroma to examine the contribution of HMEX in acidification of microenvironments in distal regions accessible to exosomes.

Micro RNAs (miRNAs) are approximately 22 nucleotides long, single stranded, non-protein-coding RNA molecules that can recognise and bind 3′-untranslated regions of mRNA, effectively blocking translation of the gene. There is increasing evidence that circulating miRNA in melanoma patients can be used in surveilling cancer progression^28^. Circulating miRNAs such as miR-210 have been used in a direct plasma assay to identify early systemic metastasis recurrence in melanoma patients^29^. There is now evidence that circulating miRNAs are not “free-floating” but are primarily packaged into exosomes^30^. miRNA can also directly regulate energy metabolism critical for cancer progression. For instance, miR-155 can upregulate glucose metabolism, leading to increased glycolysis^31^ while upregulation of miR-210 is capable of decreasing OXPHOS under non-hypoxic conditions^32^.

Proteomic analysis of melanoma exosomes has provided clues to the capacity of melanoma exosomes to redirect the host’s metabolic programming to favor the less efficient production of ATP by the anaerobic glycolytic pathway over oxidative phosphorylation (OXPHOS)^33^. However, it is unclear whether normal fibroblasts unexposed to factors from TME can also undergo metabolic reprogramming by exosomes alone. We hypothesised that miRNA contained within HMEX can drive the modulation of metabolic activities in HADF, and that exosomes serve as important vehicles for re-programming and generating an acidified, pre-metastatic microenvironment.

## Results

**Figure 1:**
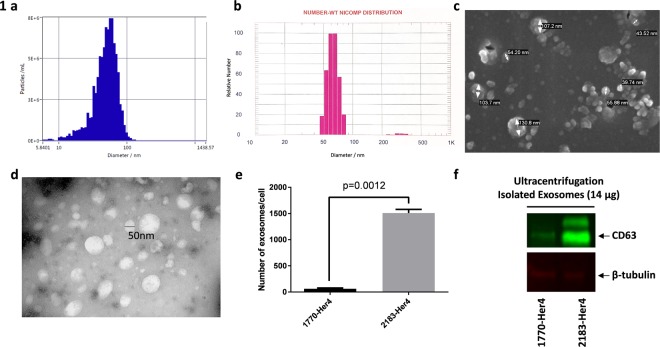
(**a**) ZetaView result of HMEX isolation (2183-Her4). Peak vesicle size is 54.4 nm. (**b**) Dynamic light scattering (DLS) result of HMEX isolation (2183-Her4). Peak vesicle size is 58 nm. (**c**) Scanning electron microscopy of HMEX isolation (2183-Her4). (**d**) TEM image of HMEX isolation (2183-Her4). The internal region of vesicles remains white, inaccessible to staining during negative staining. (**e**) Exosome count based on Exo-ELISA kit. Number of exosomes is based on a predetermined titration of CD63 antibody binding exosomes. (**f**) Western blot of HMEX. Infrared-based western blot staining with exosome-enriched marker anti-CD63 and cytoplasmic marker anti-β-tubulin antibodies. Full-length blots are presented in Supplementary Fig. S1.

Using NTA-based NanoSight, vesicles isolated by ultracentrifugation were found to have a mean size distribution of 51.0 ± 18.1 nm (Fig. 1a). Size distribution was further verified using the nanoparticle sensing technique of dynamic light scattering (DLS). NICOMP distribution analysis showed that isolated vesicle sizes have a mean diameter of 63.7 ± 7.4 nm (Fig. 1b). To visualise these particulates as discrete circular vesicles, scanning electron microscopy (SEM) was used (Fig. 1c). Transmission electron microscopy (TEM) revealed the presence of intact, nonpermeable membranous vesicles approximately 50 nm in size (Fig. 1d). These results confirmed that the physical properties of isolated vesicles are consistent with exosomes, based on size distribution^34^.

For the initial study, two melanoma cell lines 1770-Her4 and 2183-Her4 were used. One standard method of quantifying exosomes has been to use a CD63-based ELISA (EXO-ELISA) that correlates CD63 on the surface of exosomes to the number of exosomes isolated. It was observed that the two different melanoma cell lines secreted drastically different numbers of exosomes (Fig. 1e). A Western blot for CD63 expression was then performed using 14 ug protein isolated from 1770-Her4 and 2183-Her4 ultracentrifuged exosomes (Fig. 1f). CD63 expression *via* Western blot also varied dramatically between the 2 cell lines and correlated to the EXO-ELISA data. Hence, there was a need to identify melanoma cell lines that produced a substantial number of exosomes to obtain sufficient exosomes for the study.

**Figure 2:**
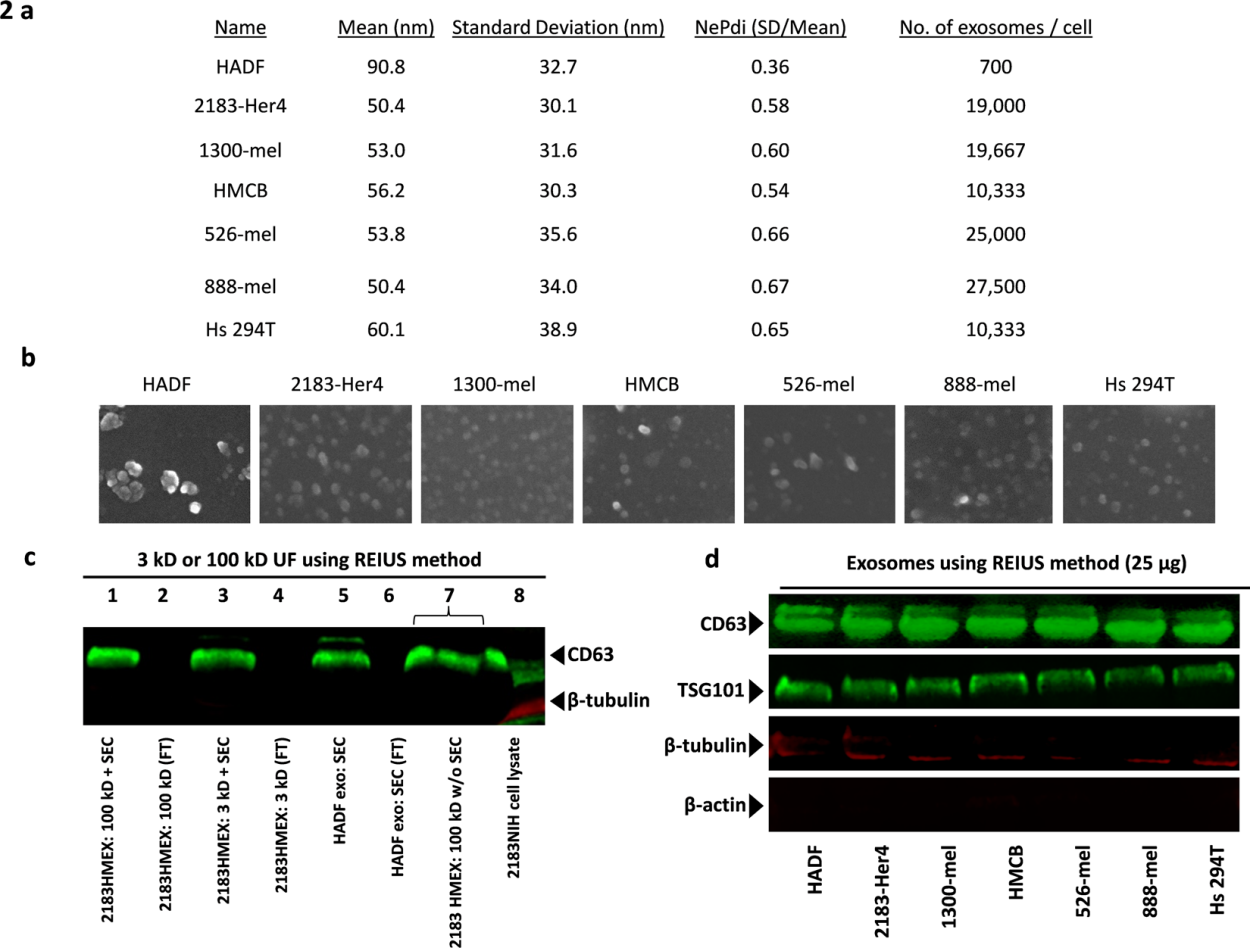
Isolation of exosomes using the REIUS method. (**a**) Physical characteristics of HADF and melanoma cell line exosomes based on NTA (Zetaview). Exosome polydispersity index (NePdi) is the ratio of standard deviation over mean exosome size based on NTA (Zetaview). (**b**) Scanning electron microscope images of HADF exosomes and six HMEX. REIUS method isolated exosomes consistently resemble circular vesicles in shape. (**c**) Infrared-based western blot staining with exosome-enriched marker anti-CD63 and cytoplasmic marker anti-β-tubulin antibodies. Lane 1: 25 µg HMEX protein following REIUS method using a 100 kDa ultrafiltration spin column. Lane 2: 25 µl flow-through following SEC of REIUS method. Absence of CD63 expression indicates no loss of exosomes following entire REIUS method. Lanes 3 and 4: same as lanes 1 and 2 except that a 3 kDa ultrafiltration spin column was used. Similar intensity of CD63 expression in lanes 1 and 3 indicate exosomes are not lost during the REIUS method when a 100 kDa spin column is used. Lanes 5 and 6: same as lanes 1 and 2 but with HADF exosomes. Lane 7: 25 µl HMEX supernatant following REIUS that did not include SEC. The increased lane width indicates presence of non-exosomal protein that would have been removed if SEC had been utilized. Lane 8: 2183-Her4 cell lysate. β-tubulin expression is only seen in this lane, indicating preparations in other lanes are not contaminated by cellular protein. (**d**) HADF exosomes and HMEX from 6 cell lines: infrared-based western blot staining with anti-CD63, anti-TSG101, anti-β-tubulin and anti-β-actin antibodies. 25 μg protein was loaded per lane. Full-length blots for are presented in Supplementary Figs S2 and S3.

In order to perform downstream applications on exosomes, another isolation method was sought that would yield a greater number of exosomes than ultracentrifugation-based isolation. Thus the REIUS method (Rapid Exosome Isolation using Ultrafiltration and Size Exclusion Chromatography) of isolating exosomes was established in our lab. This method reliably yields 6-fold higher quantities of exosomes compared to ultracentrifugation, with a mean particle size between 40 and 60 nm. Using this method, six cell lines (2183-Her4, 1300-mel, HMCB, 526-mel, 888-mel and Hs 294 T) that consistently produced substantial numbers of exosomes were identified and chosen for further study (Fig. 2a).

Isolated exosomes were observed under SEM and found to have a consistent, individually spaced, vesicular and rounded morphology (Fig. 2b). The REIUS method resulted in no detectable loss of exosomes after the SEC isolation process compared to ultrafiltration alone (Fig. 2c). The isolated exosomes had very little cytoplasmic protein contamination, as demonstrated by little β-tubulin expression (Fig. 2c,d), β-actin expression (Fig. 2d) and no detectable cyclophilin B expression (Supplementary Fig. S3b).

**Figure 3:**
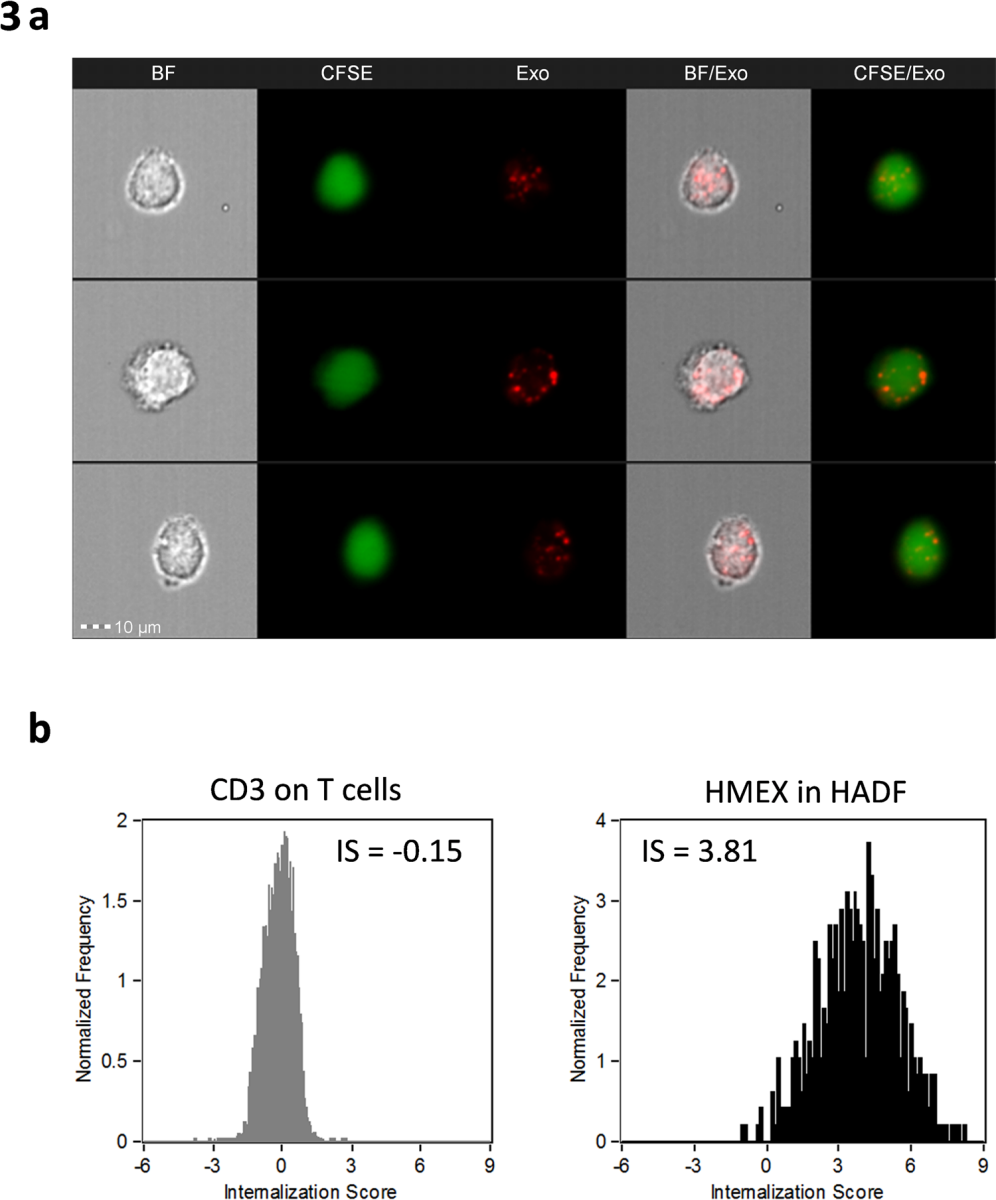
Confirmation of uptake of HMEX by HADF using ImageStream. (**a**) Representative images of the internalized exosomes were defined using Internalization feature with the Erode mask on Brightfield (BF) of 5 pixels. Examples of 3 different HADF. BF = HADF images under Brightfield microscopy. CSFE = staining of cytoplasm. exo = staining of exosomes with CVM. (**b**) Measuring internalization of HMEX by HADF. The internalization score for a target that is not expected to be internalized (CD3 on T cells) as well as one that is (HMEX in HADF).

Next, it was examined whether stromal cells such as HADF could uptake HMEX. This was confirmed by internalisation as measured by ImageStream (Fig. 3a). The Internalisation Score (IS) of HMEX by HADF was found to be 3.81, demonstrating that CellVue dye-stained melanoma exosomes internalised by HADF (Fig. 3b). Uptake of HMEX is observed in 56% of HADF after 15 minutes and 90% after 1 hour co-culture with HMEX and steadily increases to 99% over 24 hours (Supplementary Fig. S4).

**Figure 4:**
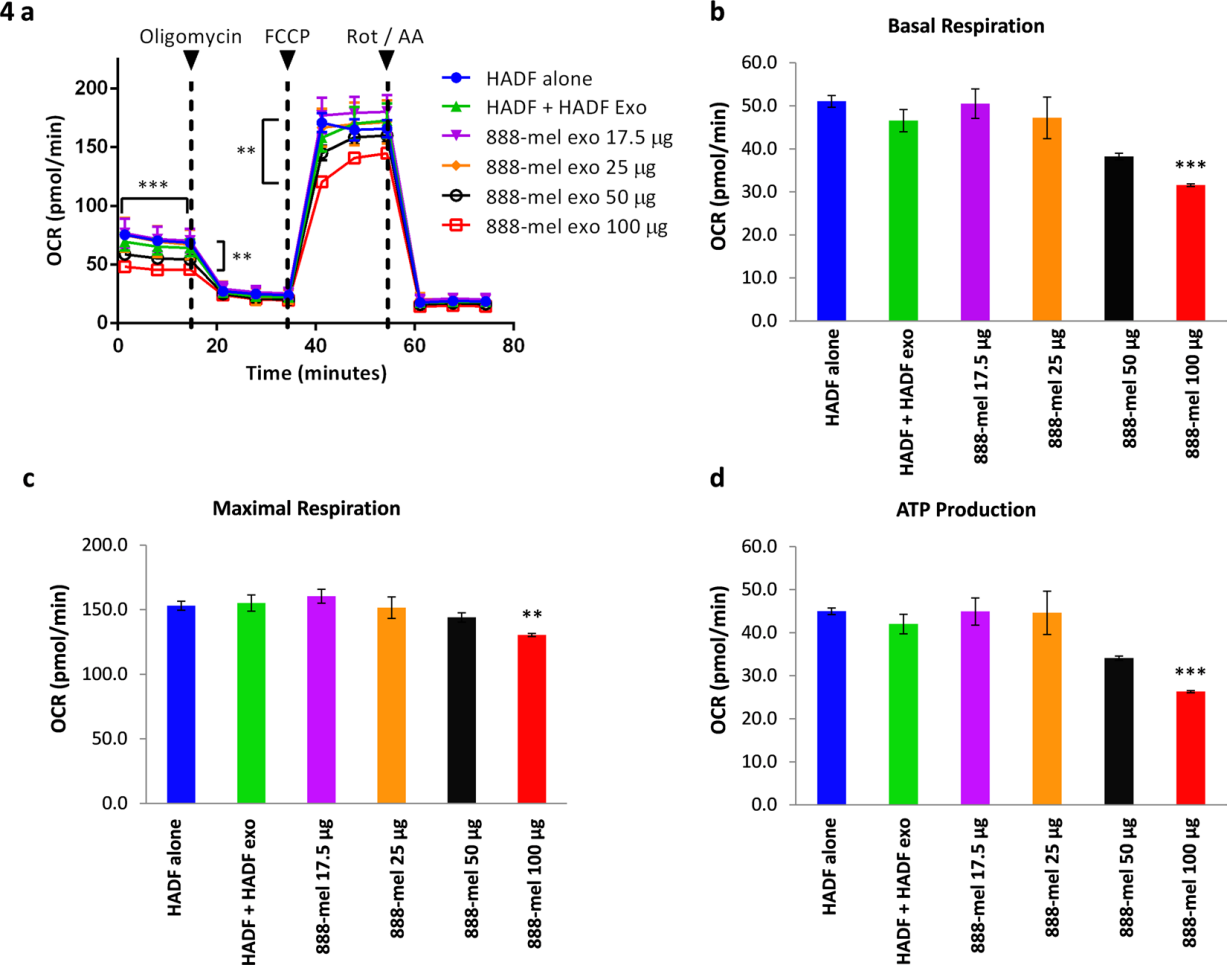
HMEX isolated through REIUS method reprograms metabolic profile of HADF to reduce mitochondria OXPHOS as demonstrated by the Mito Stress Test. HADF cells were cultured with HADF exosomes (25 µg) or HMEX (17.5 µg, 25 µg. 50 µg or 100 µg) for 18 h and examined for oxygen consumption rate (OCR) and extracellular acidification rate (ECAR). (**a**) Measuring OCR before and after addition of oligomycin, an ATP synthase (complex V) inhibitor, correlates to the mitochondrial respiration from ATP production. FCCP uncouples oxygen consumption from ATP production and the resultant spike in OCR measures maximal respiration. Rotenone and antimycin A inhibits mitochondrial respiration, demonstrating the extent of mitochondrial respiration. HADF alone: HADF in RPMI 5% exosome depleted FBS. HADF + sup: supernatant of 888-mel following ultrafiltration. HADF + HADF exo: HADF cultured with their own isolated exosomes. 888-mel exo: HADF cultured with increasing amounts of HMEX exosomes (**b**). Analysis of basal respiration of HADF following HADF exosomes or HMEX treatment based on OCR. Basal respiration is the difference in value between last rate measurement before first injection of oligomycin and non-mitochondrial respiration rate (**c**). Analysis of maximal mitochondrial respiration of HADF following HADF exosomes or HMEX treatment based on OCR. Maximal mitochondrial respiration is the difference in value between maximum rate measurement after FCCP injection and non-mitochondrial respiration. (**d**) Analysis of ATP production of HADF following HADF exosomes or HMEX treatment based on OCR. ATP production is the difference in value between highest rate measurement after oligomycin injection and minimum rate measurement after oligomycin injection.

**Figure 5:**
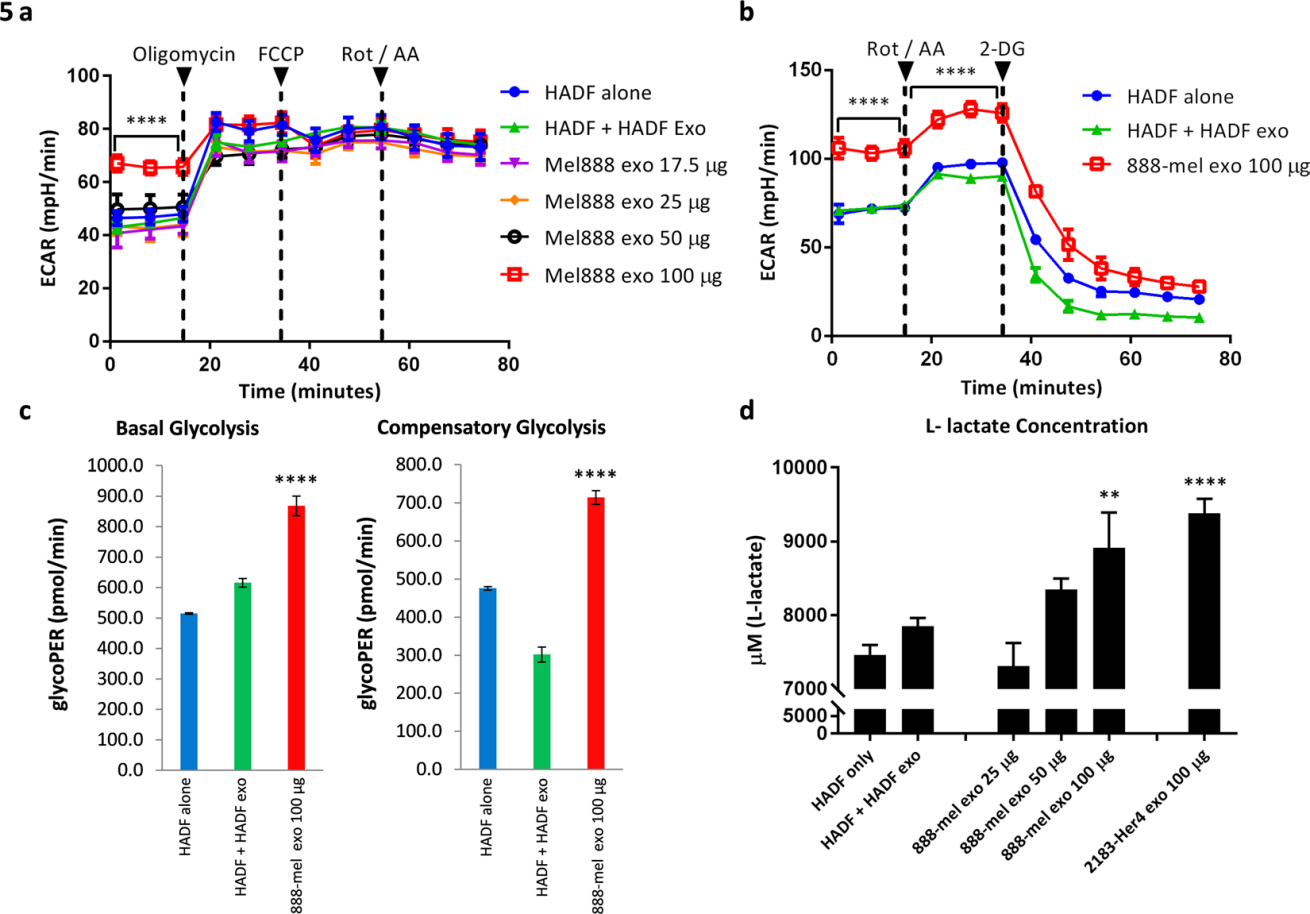
HMEX exosomes increase extracellular acidity of HADF. (**a**) HADF cells were cultured with either exosomes from HADF or HMEX for 18 h and examined for extracellular acidification rate (ECAR) during Mito Stress Test. ECAR is significantly increased in 100 μg 888-mel exosome-treated HADF compared to controls (vs HADF exosomes) (**b**). HMEX isolated through REIUS method reprograms metabolic profile of HADF to increase glycolysis, as demonstrated by the Glycolytic Rate Test. Rotenone and antimycin A inhibits mitochondrial respiration and the resultant ECAR increase correlates with the complete metabolic shift from OXPHOS to glycolysis. Addition of 2-deoxy-glucose (2-DG) inhibits glycolysis, resulting in ECAR decrease, confirming the ECAR produced in the experiment is due to glycolysis. HADF alone: HADF in RPMI 5% exosome depleted FBS. HADF + HADF exo: HADF cultured with their own isolated exosomes. 888-mel exo: HADF cultured with 100 μg 888-mel exosomes. (**c**) Basal glycolysis and compensatory glycolysis of HADF following HADF exosome or HMEX treatment, based on Glycolytic Proton Efflux Rate (glycoPER) converted from OCR and ECAR data through Seahorse XF Glycolytic Rate Assay Report. (**d**) L-lactate assay on HADF treated with HMEX isolated through REIUS method. d. L-lactate is synthesized as an end-product for glycolysis. Addition of HMEX from 888-mel (V600E mutant BRAF) and 2183 (wild-type BRAF) both significantly increase the amount of L-lactate synthesized by HADF over 18 h period compared to HADF exosomes added to HADF.

To determine whether isolated HMEX are by themselves capable of reprogramming normal stromal cells, the Seahorse Mito Stress test analysis was used to measure aerobic respiration rate in HADF, i.e. oxidative phosphorylation (OXPHOS). Since downstream analyses required large numbers of exosomes, 888-mel was chosen as it had an inherently high proliferation rate and produced the most exosomes among the six cell lines previously tested. Addition of 100 μg of HMEX were capable of downregulating the basal respiration of HADF by 32.2% (46.5 ± 2.58 pmol/min to 31.6 ± 0.30 pmol/min), maximal respiration by 15.9% (155 ± 6.29 to 130 ± 1.19 pmol/min) and ATP synthesis by 37.4% (42.0 ± 2.27 to 26.3 ± 0.25 pmol/min) (Fig. 4a–d). Moreover, HADF that were exposed to HMEX also increased extracellular acidification (ECAR) of HADF when compared to control (Fig. 5a). With increased glycolysis by HMEX being a possible mechanism of acidification, a glycolysis-specific assay (GlycoRate) was used to measure the basal and compensatory glycolysis in HADF cells (Fig. 5b). Compared to the control, HMEX exposed HADF exhibited increased basal glycolysis by 41.0% (from 616 ± 24.6 to 868 ± 45.6 mpH/min) and compensatory glycolysis by 136.8% (from 301 ± 34.4 to 714 ± 25.7 mpH/min), indicating a metabolic shift towards glycolysis (Fig. 5c). This was further confirmed by use of an L-lactate glycolysis assay to measure the byproduct of glycolysis, which increased by 13.5% (from 7854 ± 112 to 8914 ± 480 μM) in HMEX exposed HADF cells compared to control (Fig. 5d).

As the study focuses on the potential of HMEX in traversing distal regions possibly through known channels of dissemination such as blood vessels, the concentration of exosomes used in our study had to be kept physiologically relevant. The number of exosomes used in our study (100 µg) correlates with approximately 10^11^ exosomes/ml. Given that serum can carry approximately 10^12^ exosomes/ml^35^, the amount of exosomes used in this study is a physiological concentration that does not impact viability of HADF after exposure (Supplementary Fig. S5).

**Figure 6:**
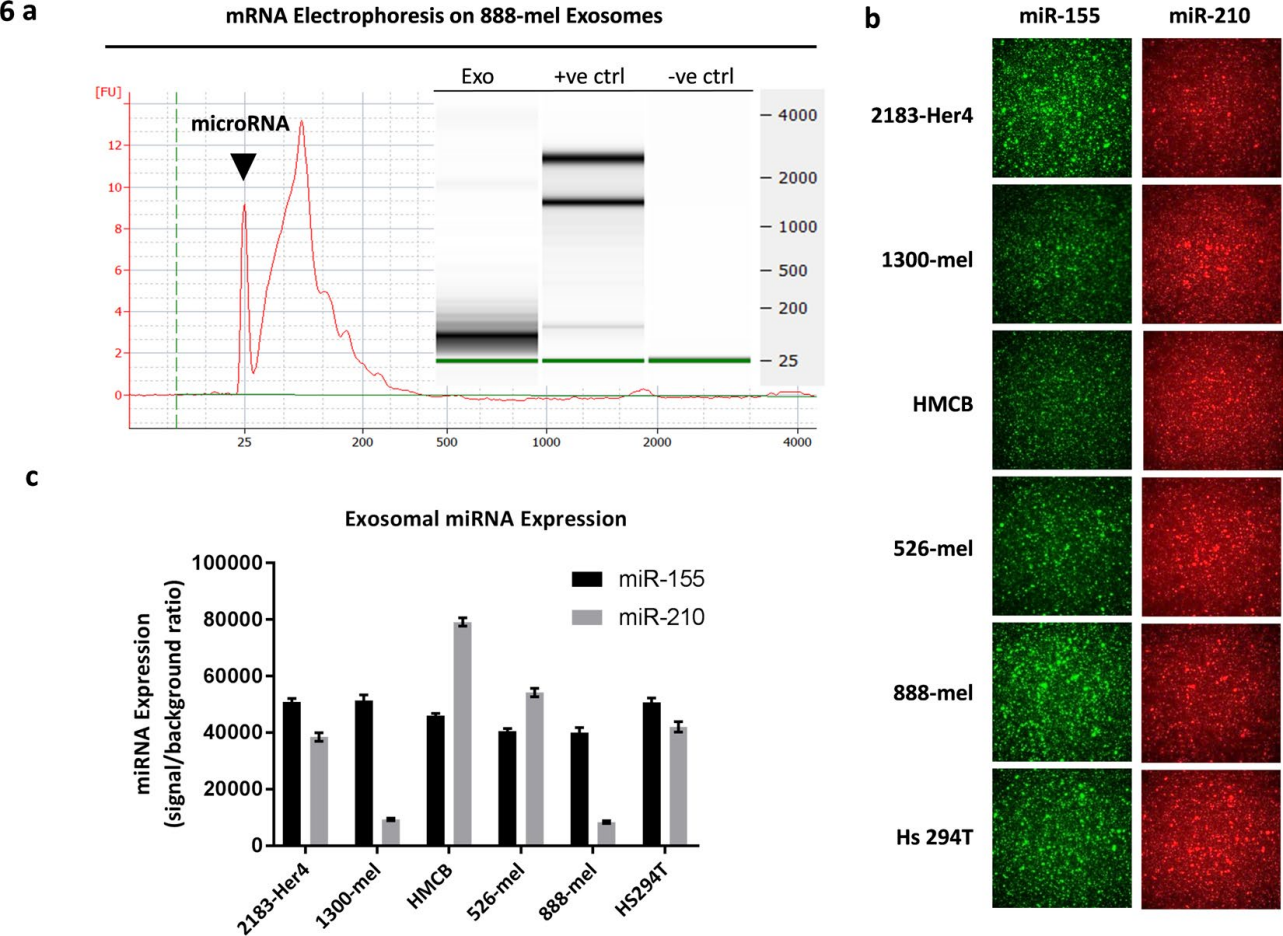
miR-155 and miR-210 is present in HMEX across six melanoma cell lines. (**a**) mRNA electrophoresis of 888-mel HMEX. A distinct peak of approximately 25 nucleotides is observed, a typical nucleotide size for miRNA. (**b**) Total Internal Reflection Fluorescence (TIRF) microscopy images of HMEX from six melanoma cell lines. (**c**) miRNA expression was correlated to total signal intensity using known standards as controls.

**Figure 7:**
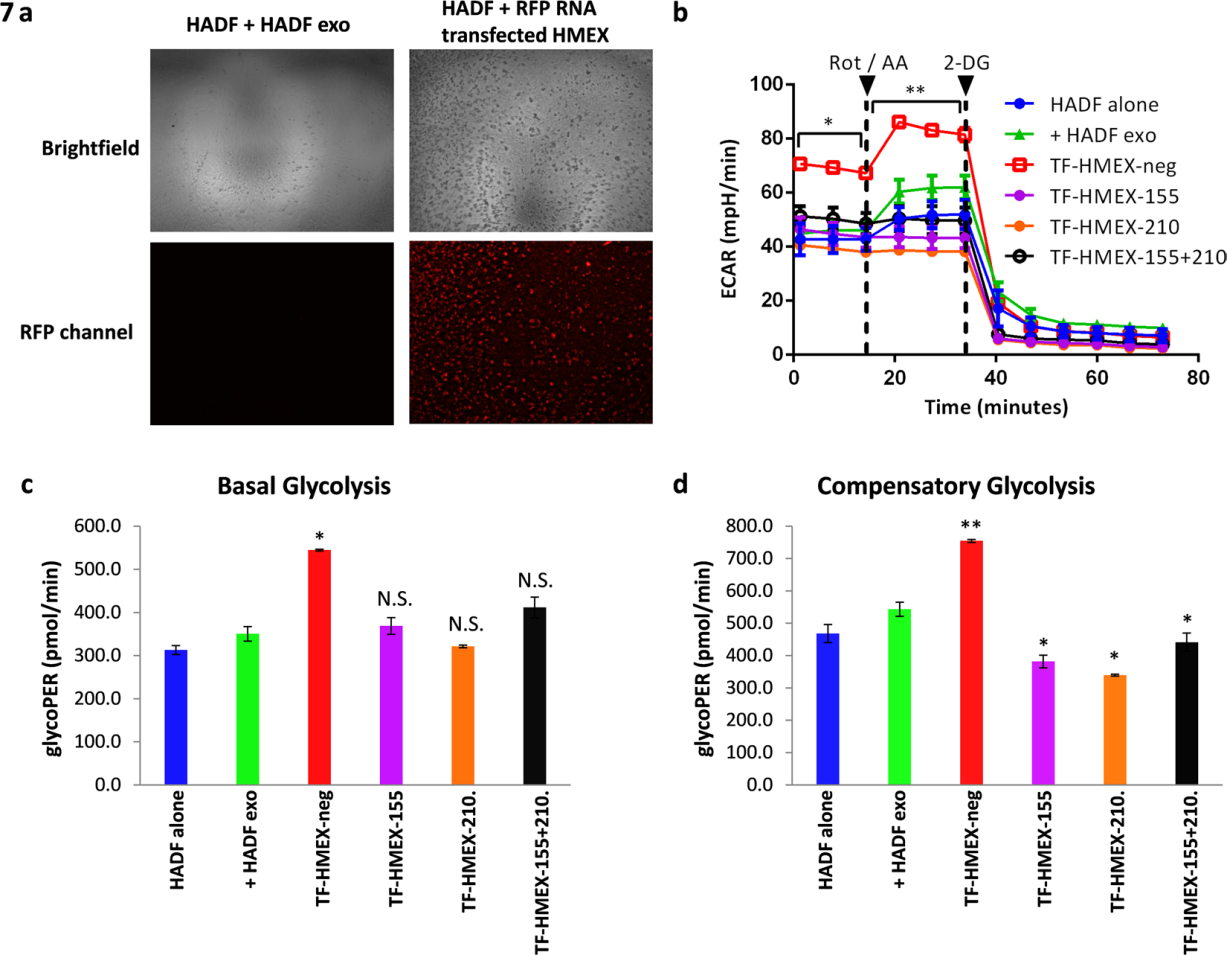
Transfected HMEX carrying miRNA inhibitors can reverse the glycolysis-driven metabolism programming in HADF. (**a**) Successful transfection of HMEX with RFP RNA and detection of RFP fluorescence compared to HADF without RFP RNA transfection. (**b**) GlycoRate Test. HADF cells were cultured with HADF exosomes ( + HADF exo), miRNA negative control (TF-HMEX-neg), HMEX transfected with miR-155 inhibitor (TF-HMEX-155), miR-210 inhibitor (TF-HMEX-210) or miR-155 and miR-210 inhibitor (TF-HMEX-155 + 210) or for 18 h and examined for extracellular acidification rate (mPER). (**c**) Basal Glycolysis. (**d**) Compensatory Glycolysis is reversed in HMEX transfected with miR-155 and miR-210 inhibitors when compared to HADF incubated with HMEX (TF-HMEX-neg).

We next determined whether this observation is due to an exosomal payload that may be unique to V600E BRAF mutant cell lines and not wild-type. However, the same observations were also true for wild-type BRAF 2183-Her4 cell line HMEX, indicating that the elevation of L-lactate is not limited to BRAF V600E mutant cell line HMEX alone. In light of previous findings, we hypothesised that microRNA was involved due to its rapid influence on cellular programming. To test this hypothesis, we examined melanoma exosomes for the presence of miRNA and observed a distinct peak at 25 nucleotide using a 2100 Bioanalyzer (Fig. 6a). A literature search yielded 2 miRNA candidates: miR-155, capable of driving metabolic reprogramming to increase glycolysis^36,37^ and miR-210, capable of metabolic reprogramming by decreasing mitochondrial OXPHOS activity^38^. To confirm the presence of these miRNAs within the exosomes, an immune-biochip technology adapted from a previously developed tCLN biochip was employed^39^. Exosomes derived from six melanoma cell lines (2183-Her4, 1300-mel, HMCB, 526-mel, 888-me and, Hs 294 T) were found to contain miR-155 and miR-210 (Fig. 6b,c). We further tested the hypothesis that the presence of these metabolically modulating miRNAs are responsible for the metabolic reprogramming observed in HADF by transfecting HMEX with miRNA inhibitors. The capacity of exosomes to carry RNA cargo into the HADF was confirmed by the red fluorescence signal in HADF incubated with exosomes transfected with red fluorescent protein RNA (Fig. 7a). Using the same technique exosomes were transfected with miRNA inhibitors specific for miR-155 and/or miR-210 (TF-HMEX) and the level of glycolysis was examined using an extracellular flux assay (Fig. 7b). Consistent with our hypothesis, TF-HMEX with miR155 inhibitor (TF-HMEX-155), miR210 inhibitor (TF-HMEX-210) and both miR155 and miR210 inhibitors (TF-HMEX-155 + 210) reversed the significant increase in glycolysis when compared to TF-HMEX with miRNA inhibitor negative control (TF-HMEX-neg). Specifically, the basal glycolysis that is significantly increased in TF-HMEX-neg by 58.8% vs control (506.6 ± 37.9 vs 350.3 ± 16.9 pmol/min) is no longer observed in TF-HMEX-155 (368.7 ± 19.4 pmol/min), TF-HMEX-210 (357.2 ± 36.0 pmol/min) or TF-HMEX-155 + 210 (411.5 ± 24.1 pmol/min) (Fig. 7c). Compensatory glycolysis by TF-HMEX was also reversed, i.e. the compensatory glycolysis that is significantly increased in TF-HMEX-neg by 38.8% vs control (754.4 ± 4.6 vs 543.2 ± 21.9 pmol/min) is no longer observed (rather, significantly decreased) in TF-HMEX-155 (381.7 ± 19.6 pmol/min), TF-HMEX-210 (339.5 ± 3.0 pmol/min) or TF-HMEX-155 + 210 (441.1.5 ± 28.5 pmol/min) compared to control (Fig. 7d). This significant reduction of glycolytic capacity in TF-HMEX treated HADF clearly demonstrated the capacity of TF-HMEX to nullify the HMEX-based glycolysis-enhancement.

Not only is increase in glycolysis by HMEX on HADF reversed after miR-155 and/or miR-210 inhibition, downregulation of OXPHOS by HMEX on HADF is also reversed when compared to TF-HMEX-neg. TF-HMEX-neg exhibited a significant decrease of 61% in basal respiration (19.5 ± 0.3 pmol/min vs 31.4 ± 2.0 pmol/min), 39.3% decrease in maximal respiration (57.5 ± 2.2 vs 94.8 ± 5.0 pmol/min) and 36.5% decrease ATP production versus control (16.9 + 1.3 vs 26.5 ± 1.0 pmol/min). The decrease was reversed in TF-HMEX-155 (Basal: 26.1 ± 1.8 pmol/min, Maximal:86.6 ± 5.9 and ATP production: 22.7 ± 1.2), TF-HMEX-210 (Basal: 24.6 ± 3.8 pmol/min, Maximal:88.1 ± 5.2 and ATP production: 21.2 ± 3.0) and TF-HMEX-155 + 210 (Basal: 30.9 ± 2.1 pmol/min, Maximal:102.6 ± 0.1 and ATP production: 24.8 ± 2.4) compared to control (Supplementary Fig. S6). We conclude that melanoma-derived exosomes are biologically active, and capable of delivering miR-155 and miR-210 to the recipient HADF, leading to a change in metabolic preference in HADF by upregulating glycolysis and downregulating OXPHOS and in these recipient stromal cells.

## Discussion

This study clearly demonstrates that HMEX and specifically its miRNAs are capable of reprograming the metabolism of stromal fibroblasts to increase aerobic glycolysis. A recent concept coined “reverse Warburg effect”, unlike its implied meaning, is rather a continuation of the “Warburg effect” by reprogrammed stromal cells (e.g. fibroblasts) favoring aerobic glycolysis to produce high energy fuels (e.g. lactate) which are then transported back for use by the cancer cells^40–42^. The finding updates our current understanding that instigators of extracellular acidification that favours conducive microenvironment or “soil” for cancer progression may not be solely confined to the specific site of cancer, and that such a metastasis-favoring niche can also be generated by normal stromal fibroblasts in distal regions that are exposed to metabolic reprogramming factors of TME such as cancer exosomes. There is greater anticipation for future research to re-examine the consequences of an acidified, immunosuppressive microenvironment created beyond the TME where cancer exosomes can interact with normal stromal fibroblasts, as exosomes have unrestricted access to the lymphatic system and blood vessels^23,37,43^.

Cancer exosomes can promote metastasis by conditioning stromal cells^15,19,43,44^. The number of cancer exosomes produced by cell lines varies. 2183-Her4 from a primary site of melanoma (subcutaneous, shoulder) expressed significantly greater quantities of exosomes than 1770-Her4 derived from a secondary metastatic site of melanoma (jejunum). It would be interesting to examine in the future whether melanoma cell lines that generate more exosomes have a more aggressive metastatic potential.

Obtaining purified, functionally-active exosomes is challenging yet critical to understanding the effects these exosomes have on the metabolic reprogramming of stromal cells. To date, studies on exosomes have dedicated substantial effort to determine a method to isolate exosomes with minimal contamination by other extracellular signaling protein^45,46^. Among the prevailing isolation methods, ultracentrifugation (including sucrose gradient fractionation method), polyethylene glycol (PEG) and SEC are three distinct methods frequently employed to isolate exosomes, each with its own advantages and limitations. In this investigation, polyethylene glycol (PEG) to precipitate lipid extracellular vesicles was avoided as PEG has traditionally been used to extract glucose metabolic enzymes and antibodies^47–49^. The use of SEC has been extensively examined for its superior capacity to eliminate common protein contaminants such as albumin^50–53^. Using the REIUS method, ultrafiltration spin columns that have been used to traditionally remove soluble factors below 100 kDa in size coupled with SEC drastically reduces extracellular protein contamination.

We investigated whether melanoma-derived exosomes could reprogram normal HADF metabolism, thus contributing to the favourable conditions of a pre-metastatic niche^54^. Our demonstration of increased glycolysis and decreased OXPHOS in normal HADF exposed to HMEX and promotion of “Warburg effect” is consistent with recent findings on the ability of tumour exosomes to reprogram stromal cells^41,55,56^.

Identifying miRNA within exosomes apart from “free-floating” miRNA and other contaminants has been a challenge for exosome miRNA-based studies. To address this challenge, we used an immuno-biochip technology developed from our previous tethered cationic lipoplex nanoparticle (tCLN) biochip^39^. The immuno-biochip identifies and captures exosomes based on exosome enriched surface proteins, such as CD63. CD63 was chosen as melanoma patients express high levels of CD63 in plasma^57^. The anti-CD63 antibodies tethered on the surface of biochip captures and binds exosomes expressing CD63, further purifying the exosome isolate when unbound particles are washed off. Through the fusion of captured exosomes with CLN containing molecular beacons by electrostatic interaction, the hybridization of molecular beacons with miRNAs can be used to measure the levels of miRNAs based on restored fluorescence signals from molecular beacons. With this immuno-biochip, we have successfully detected and measured exosomal miR-155 and miR-210 in exosomes derived from six melanoma cell lines. Exosomal miR-155 and miR-210 have been reported to favour tumour progression. Exosomal miR-155 have been implicated in breast cancer chemoresistance^58^, and exosomal miR-210 from hepatocellular carcinoma and breast cancer cells taken up by cells in the TME lead to increased angiogenesis^59,60^.

**Figure 8:**
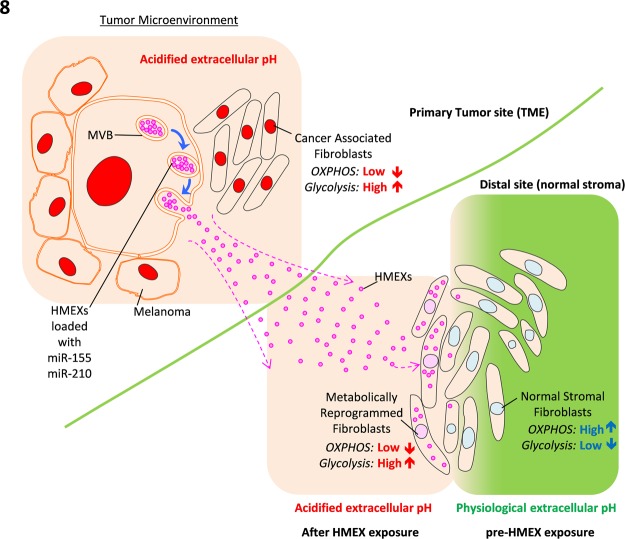
Proposed model for the modulation of pre-metastatic niche by HMEX in distal sites. HMEX generated by melanoma are taken up by normal stromal fibroblasts in distal sites where HMEX can travel through lymphatic system and blood vessels. These distal sites are slowly preconditioned into pre-metastatic sites through the metabolic reprogramming of fibroblasts by miR-155 and miR-210 delivered by HMEX to increase glycolysis and decrease the use of OXPHOS for cellular metabolism, leading to increased acidity of the microenvironment which inevitably turns into an immune sanctuary site for metastatic migration of tumor cells.

We have shown that the exosomal miR-155 and miR-210 play a pivotal role in supporting a pre-metastatic microenvironment by changing the metabolic conditioning of the normal stroma by favouring glycolysis and dampening the OXOPHOS, hence engendering the “Warburg effect.” We find that these miRNAs encased within exosomes are generated by the tumour cells, showing a clear link between tumour exosomes, miRNA and the influence of TME in distal sites (summarised in Fig. 8). miR-155 and miR-210 may not be the only molecules involved in reprogramming energy metabolism of normal stromal cells by HMEX, as there are other tumour exosomal miRNAs that were not examined^54^. This data provides new insight that the initiation of metabolic reprogramming of normal stroma begins with uptake of melanoma exosomes and offers new therapeutic opportunities to study exosomes and exosomal miRNA as targets of intervention to reduce the likelihood of metastasis in cancer patients.

## Material and Methods

### Cell culture

Human Adult Dermal Fibroblast (HADF) cell lines were obtained from the America Type Culture Collection (ATCC). BRAF wild-type melanoma cell lines (1770-Her4, 2183-Her4, 1300-mel and HMCB) and BRAF V600E mutant melanoma cell lines (526-mel, 888-mel and Hs 294 T) were either acquired from ATCC or the National Institutes of Health. Cell lines were submitted for authentication at the Genomic Shared Resource Center (Roswell Park Comprehensive Cancer Center) by short tandem repeat (STR) DNA fingerprinting using the AmpFLSTR® Identifiler® Plus PCR Amplification Kit (ThermoFisher Scientific, catalog #A26182). ATCC STR database (https://www.atcc.org/STR_Database.aspx) and DSMZ STR database (http://www.dsmz.de/fp/cgi-bin/str.html) were used for STR profiling. Cell lines HMCB and Hs 294 T were confirmed to have 100% match for both ATCC and DSMZ STR profiling. For cell lines without STR profile in the databases (1770-Her4, 2183-Her4, 1300-mel, 526-mel, and 888-mel) the STR data was saved to serve as a reference for future work. Cells were maintained in RPMI 1640 without glutamine or 4-(2-hydroxyethyl)-1-piperazineethanesulfonic acid (HEPES) but supplemented with GlutaMAX Supplement, 5–10% fetal bovine serum (FBS) (all from ThermoFisher Scientific) and 20 µg/ml Gentamicin Sulfate (Corning). All cell lines were adherents and thus passaged by washing in Dulbecco’s phosphate-buffered saline (DPBS) without calcium or magnesium (Thermo) followed by incubation with TrypLE Express Enzyme (Thermo). Prior to the last 18 hours of each experiment, cells were washed with PBS and medium was changed to RPMI/Glutamax/Gentamicin containing 5% exosome depleted FBS (exo^−^ FBS) (Gibco).

### Isolation of exosomes

Exosome isolation was modified from previously published methods^61,62^. 30 ml of cell culture supernatant was centrifuged at 300 g for 5 min (Eppendorf 5810 R, Germany) to discard the cell pellet. The supernatant was transferred to a fresh 50 ml tube and centrifuged at 3000 *g* for 15 min to remove cell debris and apoptotic bodies. The supernatant was then filtered using a 0.20 μm syringe filter (Corning, Germany) to remove remaining cells, debris and EV larger than exosomes. The resultant supernatant was isolated for exosomes using two methods: ultracentrifugation or Rapid Exosome Isolation using Ultrafiltration and Size Exclusion Chromatography (REIUS). In the first method, supernatant was ultracentrifuged at 200,000 g for 90 min at 4 °C (SW41 Ti Rotor, Swinging Bucket in Beckman Coulter L8-80M Ultracentrifuge). Following centrifugation steps, all subsequent steps were processed at 4 °C. The ultracentrifuged supernatant was carefully decanted and the pellet was suspended in an appropriate volume of PBS. For REIUS method, all steps were performed at 22 °C. The supernatant was transferred to an Amicon® Ultra-15 100 kDa (Millipore, USA) device (approximately 10 nm pore size) that serves the dual purpose of removing soluble factors that are less than 100 kDa and concentrating supernatant to approximately 200 μl using an Eppendorf 5810 R centrifuge at 3000 *g* for 15 min. The resultant concentrate was desalted and buffer exchanged by adding 1.5 ml of 0.20 μm filtered DPBS and centrifuged at 3,000 *g* for 5 min. 100 µL of filtered exosome fraction was Purified using Exo-spin™ Size Exclusion Columns (Cell Guidance Systems, USA) by centrifuging at 50 g for 60 seconds. The eluent was discarded and 200 µL of PBS was applied to each column. This was centrifuged at 50 g for 60 seconds and the eluate containing exosomes was either stored at 4 °C overnight or used immediately for experiments.

### Western blotting

Following incubation with exosomes for 24 h, cells and isolated exosomes were collected, washed with DPBS and lysed in radio-immunoprecipitation assay (RIPA) buffer with Halt protease and phosphatase inhibitors (all from Thermo Scientific). Protein concentration was determined using the Pierce BCA Protein Assay (Thermo Scientific). Equivalent amounts of protein lysates were loaded on to 4–20% Mini-PROTEAN® TGX gels (Bio-Rad) and transferred to EMD Millipore Immobilon™-FL PVDF (Fisher Scientific). Primary antibodies CD63 (HPA010088, Sigma), β-tubulin (T5201, Sigma), cyclophilin-B (43603S, Cell Signaling) and ${\rm{\beta }}$-actin (3700S Cell Signaling) were used for detection. Secondary IRDye antibodies (LI-COR) were used together with the Odyssey® Fc Imaging System (LI-COR).

### Zetaview Tracking Analysis

Nanoparticle tracking analysis (NTA) was performed on exosomal samples using ZetaView (Particle Metrix, USA). The samples were run at 25 degrees using 0.20 μm filtered DPBS as a diluent. Size and concentration of vesicles were measured using the ZetaView Nanoparticle Tracking Analyzer (Particle Metrix, Germany) with a 488 nm laser light source. For video acquisition, a shutter speed of 600 and a frame rate of 60 were used and the sensitivity was set according to the system’s software guidance algorithms. Before measurements were taken, accuracy of the ZetaView was verified by measuring with 100 nm standard beads. Samples were diluted in PBS with a dilution factor of 1:1000–1:5000 to achieve a particle count in the range of 200–500.

### Dynamic light scattering

Dynamic light scattering (DLS) analyses on purified exosomes were performed using NICOMP 380 ZLS (Particle Sizing System-NICOMP, Port Richey FL). The samples were run at 25 °C using 0.20 μm filtered DPBS as a diluent. DLS correlation data were averaged over 5 min/cycle for 2 cycles. Number-weighted analysis was used for exosome size determination.

### Transmission electron microscopy (TEM)

Negative staining technique was employed to visualise the exosomes. An enriched exosome suspension in filtered DPBS was dispensed on carbon-coated electron microscopy grids on parafilm and left to absorb for 10 min at room temperature then transferred to a drop of Uranyless® solution for 1 min and left to air dry. Excess stain was blotted away. Imaging was performed using a JEOL 100CX II transmission electron microscope (TEM) at 100 kV.

### Scanning Electron Microscopy (SEM)

For scanning electron microscopy, cells were fixed in in 2% EM grade glutaraldehyde (Electron Microscopy Sciences, USA) for 90 mins. Post fixation, cells were collected by syringe-passage onto 0.1 μm pore 13 mm polycarbonate track-etched membrane filters (Whatman, USA). Washing and fixation were done through the filter as follows: 5 ml 2.5% glutaraldehyde in PBS allowing rest of 10 mins; 10 ml PBS rest 10 mins; 5 ml 30% v/v, 50% v/v, 70% v/v, 90% v/v ethanol in water 5 mins each; 5 ml 100% ethanol twice for 5 mins each. Samples were then dried with hexamethyldisilazane (HMDS, 5 ml, 5 mins). Filters were removed, air dried, and coated with evaporated carbon at high vacuum (Denton 502 evaporator). Cells were imaged with a Hitachi SU70 FESEM at 20 KeV using combined signals from a conventional Everhart-Thornley detector (adjusted to maximise backscattered electron component) and in-lens secondary electron detector.

### Measurement of exosome quantity using ExoELISA-ULTRA kit

ExoELISA-ULTRA (System Biosciences Inc., USA) was used in accordance to manufacturer’s instructions (https://www.systembio.com/wp-content/uploads/Manual_ExoELISA-ULTRA-1.pdf). Briefly, standards and exosome samples are incubated on the provided micro-titer plate for 1 h at 37 °C, washed 3 times with 100 μl of wash buffer, and incubated with CD63 primary antibody for 1 hour. Three washes are performed for 5 minutes each with 100 μl of wash buffer followed by addition of 50 μl of secondary antibody diluted 1:5,000 in blocking buffer for 1 hour incubation with shaking. The plate is again washed 3 times with 100 μl of wash buffer before addition of TMB ELISA provided by the kit and incubated at room temperature for 15 minutes with shaking followed by addition of 50 μl of stop buffer and read immediately at 450 nm using a spectrophotometric plate reader (Synergy HTX Multi-Mode Reader, BioTek, USA). Number of exosomes were extrapolated from standard curve and was divided by the number of cells to obtain the number of exosomes per cell.

### Labelling and coculture of melanoma exosomes with fibroblasts for ImageStream

Exosomes from melanoma cell lines were isolated using REIUS method as described above. The resultant exosome-enriched pellet was suspended in 0.20 µm-filtered PBS or diluent C for labelling using CellVue Maroon (CVM, PTI Research Inc, USA) to yield an exosome isolate. For labelling of exosomes with CVM, an aliquot of CVM in ethanol was mixed with exosomes resuspended in diluent C for 5 min at room temperature. CVM-labelled exosomes were then diluted in 1:20 volume of 1% exosome-depleted FBS RPMI with 1% exo^−^ FBS. A 5 kDa molecular weight cut-off (MWCO) spin column (Millipore, USA) was used to remove and separate unbound free CVM dye. HADF cells were labelled with Carboxyfluorescein succinimidyl ester (CFSE, Invitrogen, USA) to a final concentration of 1 µM for 20 min at 37 °C. The cells were washed with PBS prior to use. CSFE-labelled HADF cells were then exposed to CVM-labelled exosomes resuspended in RPMI with 1% exo^−^ FBS for 10 min. Cells were again washed with PBS, trypsinized and resuspended in RPMI with 1% exo^−^ FBS for ImageStream analysis.

### ImageStream data acquisition and analysis

Cell images were acquired using an ImageStream MK-II Imaging Flow Cytometer (MilliporeSigma). 10,000 images were analysed using ImageStream Data Exploration and Analysis Software (IDEAS). Spectral compensation was digitally performed. The Internalization Score (IS) is defined as the ratio of the intensity inside the cell to the intensity of the entire cell. The higher the score, the greater the concentration of intensity inside the cell. The ratio is mapped to a log scale to increase the dynamic range to values between [-inf, inf]. Cells with a high degree of internalization typically have positive scores while cells with little internalization have negative scores. Cells with scores around 0 have a mix of internalization and membrane intensity. The inside of the cells was defined by eroding the default brightfield mask (M01) by 5 pixels. By applying the thus-defined mask to the analysis of the fluorescence imagery of interest (CD3 or CVM), the internalization score was calculated on an individual cell basis.

### Detection of exosomal miRNAs via immuno-biochip technology

An immuno-biochip technology was used to capture exosomes and quantify intravesicular miR-155 and miR-210. A 15 nm gold coated cover glass was first coated with the mixture of methyl-polyethylene glycol-thiol (PEG200, MW = 200 g/mol, ThermoFisher Scientific, Rockford, IL, 26132) and biotinylated-polyethylene glycol-thiol (biotin-PEG1000, MW = 1000 g/mol, Nanocs, Boston, MA, PG2-BNTH-1K) at molar ratio of 3:1 and the concentration of 10 mM in PBS by incubation at room temperature for 1 h. After unbound PEG was washed off by PBS, 50 ng/mL NeutrAvidin (ThermoFisher Scientific, 31000) was added to react with biotin for 1 h at room temperature. The anti-CD63 antibody was tethered on the surface of the biochip through avidin-biotin interaction. Then exosomes were added at 8 × 10^10^ exosomes/mL and incubated on the biochip for 1 h at room temperature to allow the capture of CD63-expressing melanoma exosomes. After unbound exosomes were washed off by PBS, we added cationic lipoplex nanoparticles (CLN) containing molecular beacons (MBs) that detect miR-155 and miR-210. The CLN/exosome fusion through electrostatic interaction led to the hybridization of exosomal miR-155 and miR-210 to MBs and restored the fluorescence of MBs. Sequences of molecular beacon probes used are [6FAM]CGCGATC[+A]CC[+C]CT[+A]TC[+A]CG[+A]TT[+A]GC[+A]TTAAGATCGCG[BHQ1] (miR-155) and [Cyanine5]CGCGATC[+T]CA[+G]CC[+G]CT[+G]TC[+A]CA[+C]GC[+A]CAGGATC-GCG[BHQ3] (miR-210), where [+A], [+T], [+G] and [+C] represent the locked nucleic acids. Total internal reflection fluorescence (TIRF) microscopy was used to detect fluorescent signals. The averaged fluorescence intensities of a total of 100 images were used as the expression of exosomal miR-155 and miR-210.

### Extracellular flux assays

For all extracellular flux assays, cells were plated at a density of 4 × 10^5^ cells per well in Seahorse XF96 cell culture microplates (Agilent Technologies, USA) the day prior to the assay. The assay plates were washed three times with assay media using the XF Prep Station and incubated at 37 °C without CO_2_ for 45 min prior to performing the assay on the Seahorse Bioscience XFe96 (Agilent Technologies, USA). The Mitochondrial Stress Test was performed in XF Base Media containing 10 mM glucose, 1 mM sodium pyruvate and 2 mM L-glutamine and the following inhibitors were added at the final concentrations: oligomycin (2 µM), carbonyl cyanide 4-(trifluoromethoxy)phenylhydrazone (FCCP) (1.5 µM) and rotenone/antimycin A (0.5 µM each). The Glycolytic Rate Assay was performed in XF Base Media without phenol red containing 5 mM HEPES, 10 mM glucose, 1 mM sodium pyruvate, and 2mM L-glutamine and the following inhibitors were added at the final concentrations: Rotenone/Antimycin A (0.5 µM each) and 2-deoxy-glycose (50 mM). Results were analyzed using WAVE version 2.6.0.31 (Agilent Technologies, USA).

### Transfection of miRNA inhibitors

Ready-to-use, human miRNA inhibitors for miR-155, miR-210 and miRIDIAN miRNA hairpin inhibitor negative control (#1) were purchased from Dharmacon, USA. 20 nM of negative control, miR-155 and/or miR-210 inhibitors were used during transfection of HMEX. Exosomes isolated using the REIUS method were incubated with Exo-Fect reagent (System Biosciences Inc.) with either miRNA inhibitors at 37 °C for 10 min or Red fluorescent protein (RFP) RNA positive control provided with kit and placed on ice for 30 min followed by washing three times with PBS using 100 kDa Amicon Ultrafiltration columns. Cells were observed using EVOS-FL imaging system for fluorescence. Successful direct transfection was confirmed using RFP fluorescence and the transfected exosomes were directly used for further experiment.

### Lactate assay

3 μl of culture media were collected 48 h after co-incubation of HADF with exosomes and were incubated with 50 μl of Assay Reaction Master Mix for 30 min at 22 °C (L-Lactate Assay Kit, Cayman Chemical, USA). Lactate concentrations in the various samples were quantified by absorbance at 450 nm and values were normalised based on cell number.

### miRNA QC

Quantitative assessment of the purified total RNA was accomplished using a Qubit Broad Range RNA kit (Thermofisher). The RNA was further evaluated qualitatively by a 2100 Bioanalyzer (Agilent technologies).

### Statistical analysis

Student’s t-test was used for statistical analysis, with P < 0.05 defined as significant. All experiments were done in triplicate, at least three separate times. All numerical values represent the mean ± S.E. Number of asterisks denote minimum statistical significance, i.e. *p < 0.05, **p < 0.01, ***p < 0.005 and ****p < 0.001.

## Electronic supplementary material

Supplementary Figures

## Data Availability

Data sharing not applicable to this article as no datasets were generated or analysed during the current study.
