# Publisher Correction: Metabolic reprogramming of stromal fibroblasts by melanoma exosome microRNA favours a pre-metastatic microenvironment

**DOI:** 10.1038/s41598-018-37179-1

**Published:** 2019-03-15

**Authors:** Shin La Shu, Yunchen Yang, Cheryl L. Allen, Orla Maguire, Hans Minderman, Arindam Sen, Michael J. Ciesielski, Katherine A. Collins, Peter J. Bush, Prashant Singh, Xue Wang, Martin Morgan, Jun Qu, Richard B. Bankert, Theresa L. Whiteside, Yun Wu, Marc S. Ernstoff

**Affiliations:** 1 Department of Medicine, Roswell Park Comprehensive Cancer Center, Buffalo, NY USA; 2 Department of Biomedical Engineering, Jacobs School of Medicine & Biomedical Sciences, University at Buffalo, The State University of New York, Buffalo, NY USA; 3 Flow and Image Cytometry Shared Resource, Roswell Park Comprehensive Cancer Center, Buffalo, NY USA; 4 Department of Cell Stress Biology, Roswell Park Comprehensive Cancer Center, Buffalo, NY USA; 5 Department of Neurosurgery, Roswell Park Comprehensive Cancer Center, Buffalo, NY USA; 6 Immune Analysis Facility, Center for Immunotherapy, Roswell Park Comprehensive Cancer Center, Buffalo, NY USA; 7 South Campus Instrumentation Center, University at Buffalo, The State University of New York, Buffalo, NY USA; 8 Genomics Shared Resource, Roswell Park Comprehensive Cancer Center, Buffalo, NY USA; 9 New York Center of Excellence in Bioinformatics and Life Sciences, Buffalo, NY USA; 10 Department of Biostatistics and Bioinformatics, Roswell Park Comprehensive Cancer Center, Buffalo, NY USA; 11 Department of Microbiology and Immunology, Jacobs School of Medicine & Biomedical Sciences, University at Buffalo, The State University of New York, Buffalo, NY USA; 12 Department of Pathology, Immunology and Otolaryngology, University of Pittsburgh School of Medicine and UPMC Hillman Cancer Center, Pittsburgh, PA USA

Correction to: *Scientific Reports* 10.1038/s41598-018-31323-7, published online 27 August 2018

In the original version of this Article, the author Shin La Shu was incorrectly indexed. This error has now been corrected.

