# Supplementary material for: Metabolic reprogramming of stromal fibroblasts by melanoma exosome microRNA favours a pre-metastatic microenvironment
Source: Sci Rep. 2018 Aug 27;8:12905. doi: 10.1038/s41598-018-31323-7 (PMC6110845; doi:10.1038/s41598-018-31323-7)

Supplementary Figures for

Metabolic reprogramming of stromal fibroblasts by melanoma exosome microRNA favours a pre-metastatic microenvironment

Shin La Shu[a], Yunchen Yang[b], Cheryl L. Allen[a], Orla Maguire[c], Hans Minderman[c], Arindam Sen[d], Michael J. Ciesielski[e], Katherine A. Collins[f], Peter J. Bush[g], Prashant Singh[h], Xue Wang[i], Martin Morgan[j], Jun Qu[h], Richard B. Bankert[k], Theresa L. Whiteside[l], Yun Wu[b], Marc S. Ernstoff*[a]

[a]Department of Medicine, Roswell Park Comprehensive Cancer Center, Buffalo, NY
[b]Department of Biomedical Engineering, Jacobs School of Medicine & Biomedical Sciences, University at Buffalo, The State University of New York, Buffalo, NY
[c]Flow and Image Cytometry Shared Resource, Roswell Park Comprehensive Cancer Center, Buffalo, NY
[d]Department of Cell Stress Biology, Roswell Park Comprehensive Cancer Center, Buffalo, NY
[e]Department of Neurosurgery, Roswell Park Comprehensive Cancer Center, Buffalo, NY
[f]Immune Analysis Facility , Center for Immunotherapy, Roswell Park Comprehensive Cancer Center, Buffalo, NY
[g]South Campus Instrumentation Center, University at Buffalo, The State University of New York, Buffalo, NY
[h]Genomics Shared Resource, Roswell Park Comprehensive Cancer Center, Buffalo, NY
[i]New York Center of Excellence in Bioinformatics and Life Sciences, Buffalo, NY
[j]Department of Biostatistics and Bioinformatics, Roswell Park Comprehensive Cancer Center, Buffalo, NY
[k]Department of Microbiology and Immunology, Jacobs School of Medicine & Biomedical Sciences, University at Buffalo, The State University of New York, Buffalo, NY
[l]Department of Pathology, Immunology and Otolaryngology, University of Pittsburgh School of Medicine and UPMC Hillman Cancer Center, Pittsburgh, PA

***Corresponding author:**
Marc S. Ernstoff
Email: Marc.Ernstoff@RoswellPark.org

Supplementary Fig. S1: Full length blot of Fig. 1f

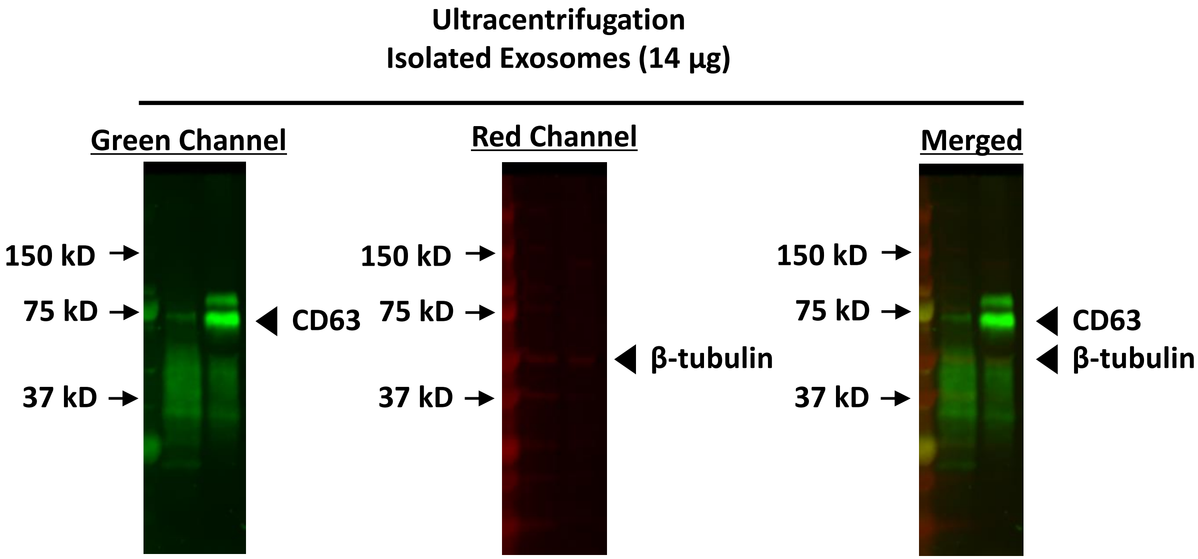

CD63 : Green Channel
β-tubulin : Red Channel

Supplementary Fig. S2 : Full length blot of Fig. 2c (Fig. S2a) and Fig. 2d (Fig. S2b)

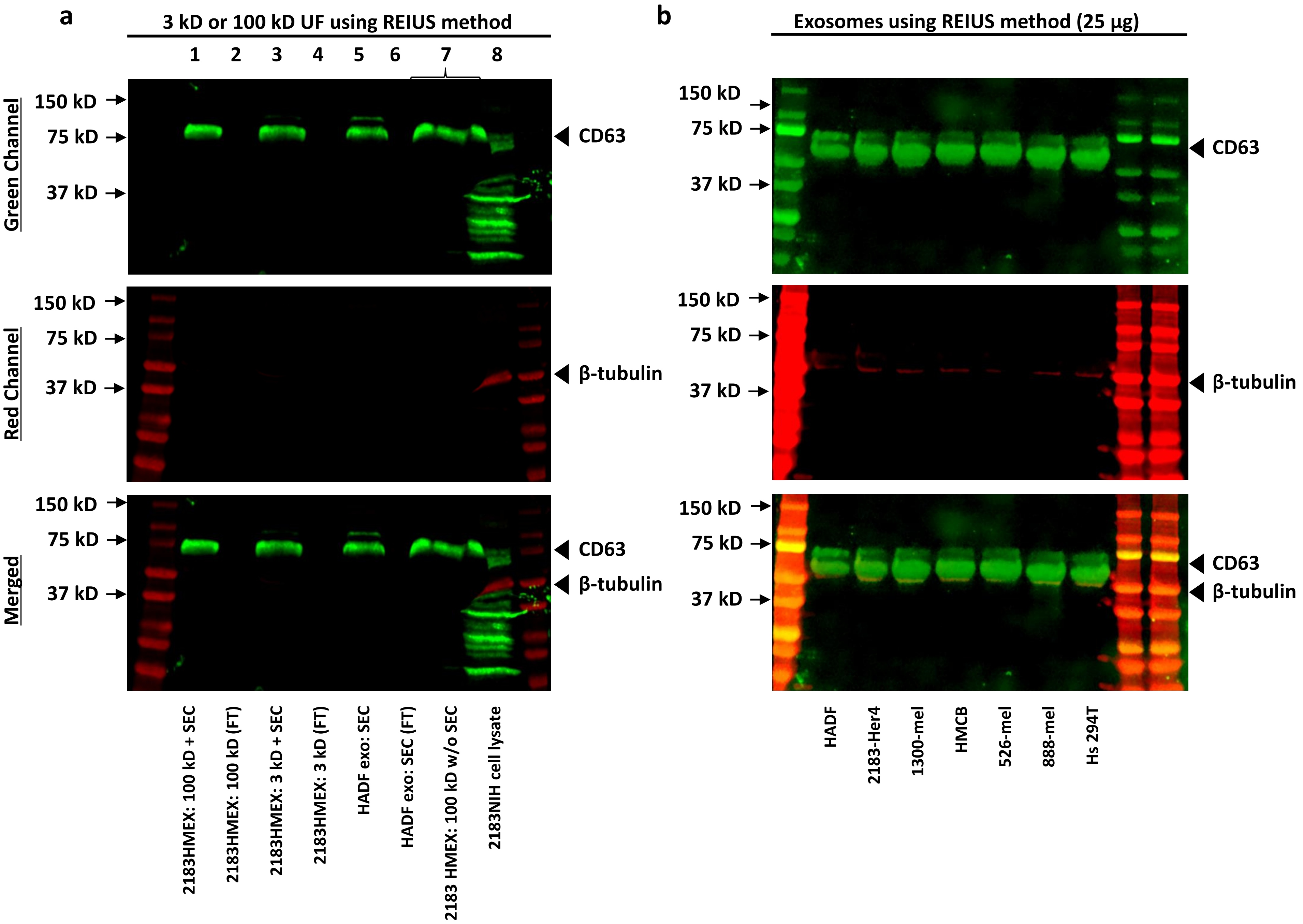

Supplementary Fig. S3 : Full length blot of TSG101 and β-actin in Fig 2d (Fig. S3a) and Fig. S3b

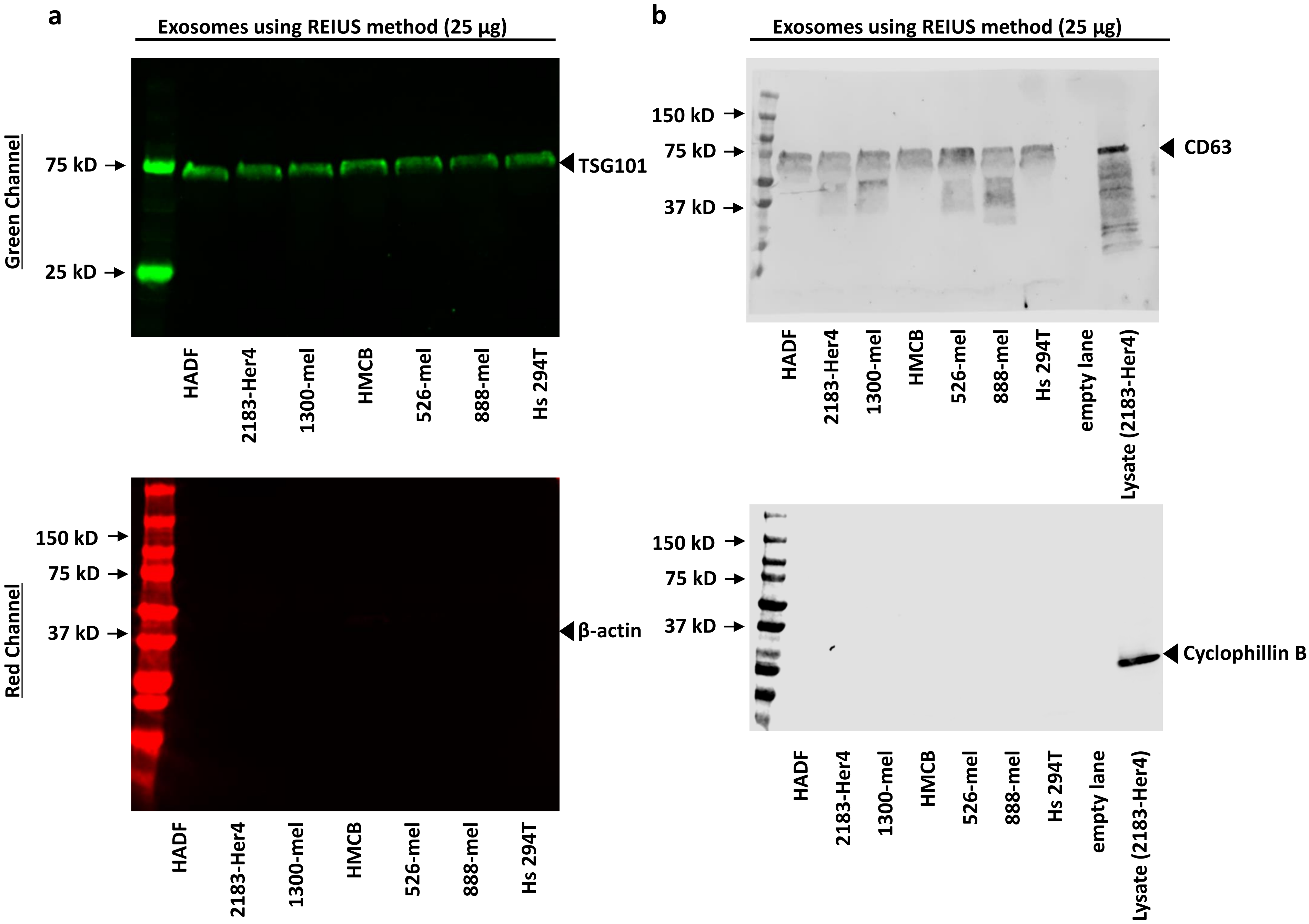

Supplementary Fig. S4

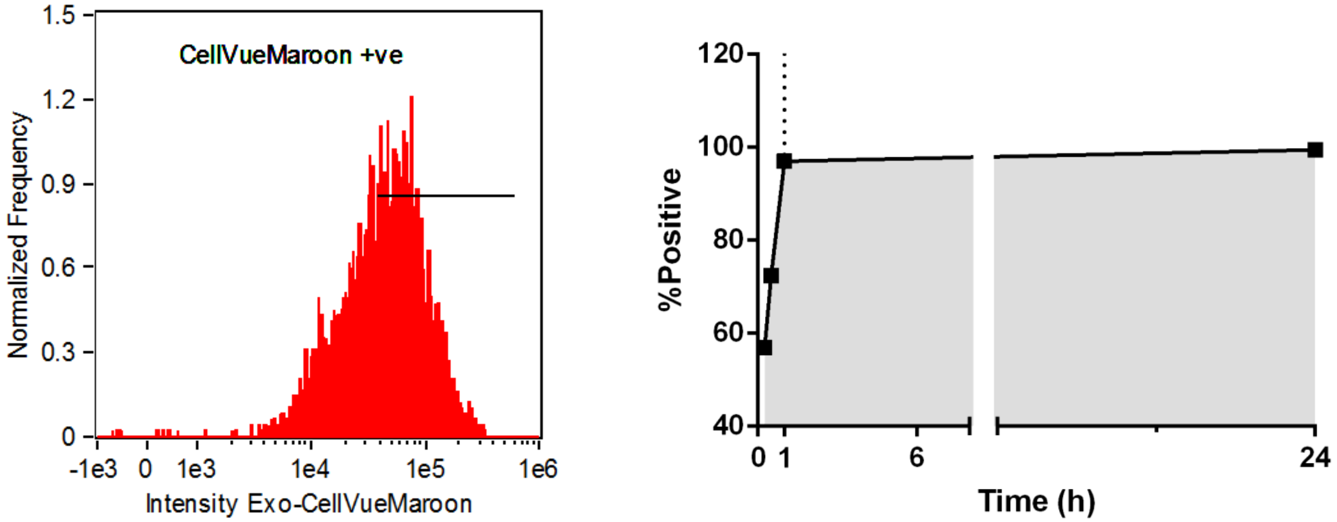

Supplementary Fig. S5

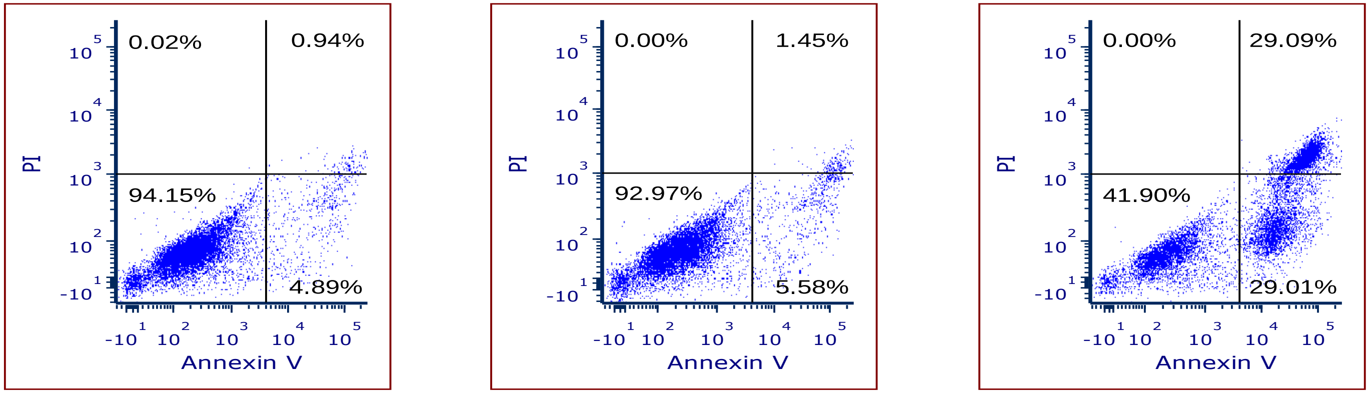

HADF alone
(No exosome coincubation)

HADF with HMEX from 888-mel
(100 ug, 24h coincubation)

Positive Control

Supplementary Fig. S6

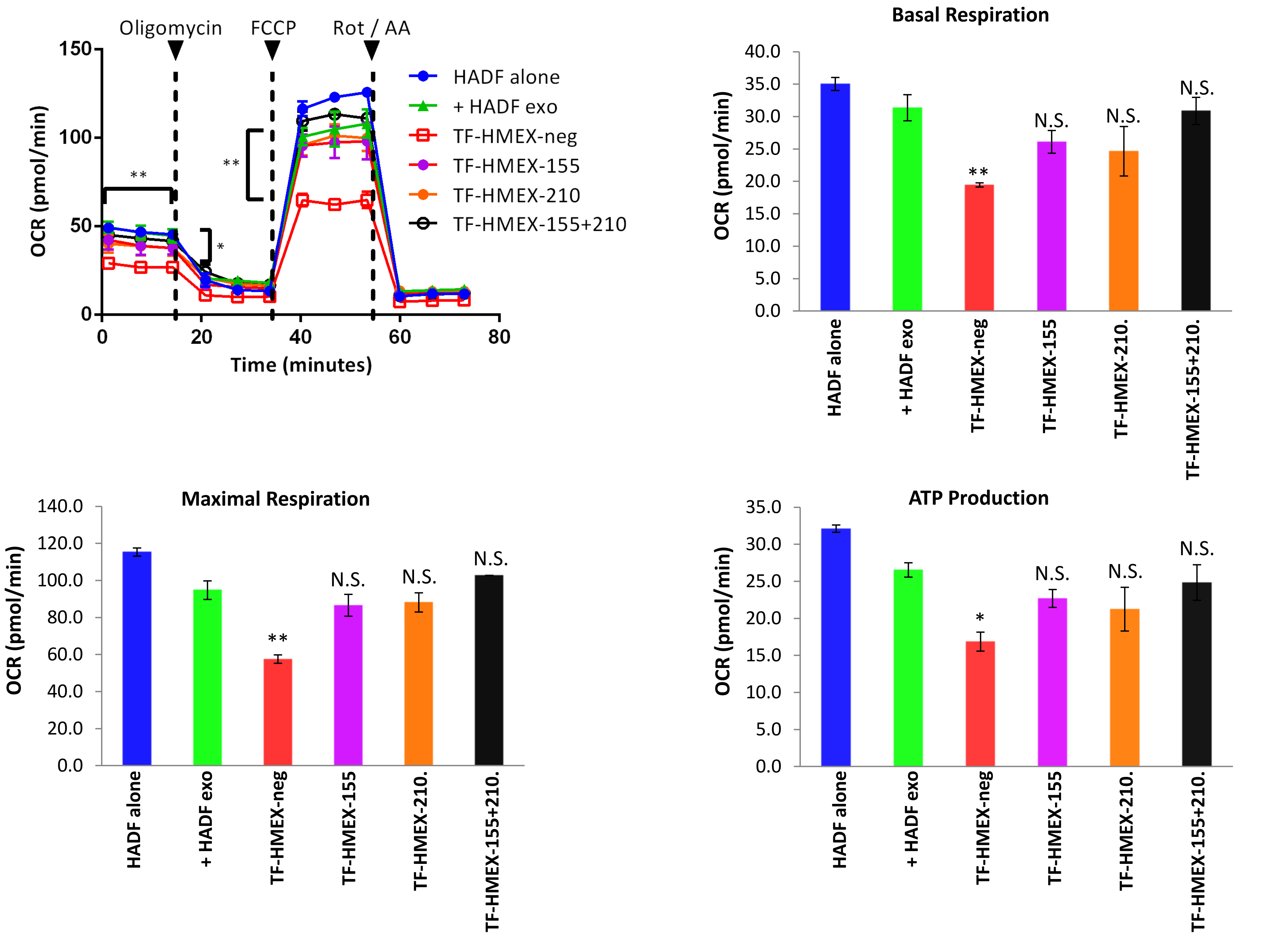

Supplement: Supplementary file 1 — Supplementary Figures [file 41598_2018_31323_MOESM1_ESM.pdf]
